# Multicrystal data collection at the VMXm beamline at Diamond Light Source

**DOI:** 10.1063/4.0000994

**Published:** 2025-10-27

**Authors:** Anna J Warren, Jose Trincao, Adam D Crawshaw, Graham Duller, Gwyndaf Evans

**Affiliations:** 1 Diamond Light Source, Didcot, Oxfordshire, United Kingdom; 2 Rosalind Franklin Institute, Didcot, Oxfordshire, United Kingdom

## Abstract

Determining the structure of a protein is essential for understanding its function. However, X-ray crystallography becomes increasingly difficult as the diffracting power of crystals decreases with a decrease in crystal size. This challenge is further exacerbated by the fact that more complex targets tend to crystallize on smaller scales, and efforts to produce larger crystals often fail. Over recent years, serial crystallography techniques at synchrotrons and X-ray free electron lasers (XFEL) have been developed to enable structure determination from smaller crystals and to carry out time-resolved experiments.^1^ Unfortunately, these methods require large sample quantities, which can be difficult, costly, and time-consuming to produce, particularly for novel systems where little prior information is known. For crystals smaller than 300 nm, micro-electron diffraction (microED) has emerged as a solution for structure determination.^2^ However, it can be challenging to confirm that a sample is of the correct size for these experiments, and often, samples are too large, necessitating focused ion beam milling to achieve the required sample thickness.^3, 4, 5^

The Versatile Macromolecular Crystallography Microfocus (VMXm)^6^ beamline was specifically designed to address these challenges by enabling rotation data collection from samples smaller than 20 μm, requiring only minimal sample volumes. This has been achieved through novel mounting of crystals on cryo-electron microscopy grids, blotting away excess liquid,^7^ conducting data collections in vacuum, and matching the beamsize to the crystal size. These strategies limit the background scatter, allowing weak signal from the micro/nanocrystals to be detected. An additional advantage comes from collecting diffraction data at higher X-ray energies (∼21 keV) to exploit photoelectron escape, extending the lifetime of the crystals in the beam.^8, 9^ To date, successful X-ray diffraction measurements have been performed on protein crystals as small as ∼1.2 μm and chemical crystallography samples down to 800 nm.

In this work we will present the beamline, and the novel strategies adopted to obtain multicrystal data from micro- and nanocrystals. In particular we will focus on comparisons between data collected on more traditional synchrotron beamlines, as well as XFELs to highlight the impact these strategies have on the production of high quality diffraction data.

## References

[1] Chapman, H. N., *et al*. (2011). Nature 470, 73–77. 10.1038/nature09750 21293373 PMC3429598

[2] Acehan, D., *et al*. (2024). Cell Reports Physical Science 5, 102007. 10.1016/j.xcrp.2024.102007 PMC11271257 39055735

[3] Duyvesteyn, H. M. E., *et al*.. (2018). Proceedings of the National Academy of Sciences 115, 9569–9573. 10.1073/pnas.1809978115 PMC6156647 30171169

[4] Li, X., *et al*. (2018). Biophysics Reports 4, 339–347. 10.1007/s41048-018-0075-x 30596142 PMC6276065

[5] Martynowycz, M. W. & Gonen, T. (2021). STAR Protocols 2, 100686. 10.1016/j.xpro.2021.100686 34382014 PMC8339237

[6] Warren, A. J., *et al*. (2024). 31, 1593–1608. 10.1107/S1600577524009160 PMC11542661 39475835

[7] Crawshaw, A. D., *et al*. (2021). Jornal of Visualized Experiments 172, e62306.

[8] Storm, S. L. S., *et al*. (2021). IUCrJ 8, 896–904. 10.1107/S2052252521008423 PMC8562668 34804543

[9] Storm, S. L. S., *et al*. (2020). IUCrJ 7, 129–135. 10.1107/S2052252519016178 PMC6949606 31949913

